# The allosteric activation mechanism of a phospholipase A_2_-like toxin from *Bothrops jararacussu* venom: a dynamic description

**DOI:** 10.1038/s41598-020-73134-9

**Published:** 2020-10-01

**Authors:** Antoniel A. S. Gomes, Fabio F. Cardoso, Maximilia F. Souza, Cristiano L. P. Oliveira, David Perahia, Angelo J. Magro, Marcos R. M. Fontes

**Affiliations:** 1 Departamento de Biofísica e Farmacologia, Instituto de Biociências, Universidade Estadual Paulista (UNESP), Botucatu, SP Brasil; 2 Laboratoire de Biologie et de Pharmacologie Appliquée, École Normale Supérieure Paris Saclay, UMR 8113, CNRS, 4 Avenue des Sciences, 91190 Gif-sur-Yvette, France; 3 Departamento de Física Experimental, Instituto de Física, Universidade de São Paulo (USP), São Paulo, SP Brasil; 4 Departamento de Biotecnologia e Bioprocessos, Faculdade de Ciências Agronômicas, Universidade Estadual Paulista (UNESP), Botucatu, SP Brasil; 5 Instituto de Biotecnologia, Universidade Estadual Paulista (UNESP), Botucatu, SP Brasil

**Keywords:** Molecular conformation, Molecular modelling, SAXS

## Abstract

The activation process of phospholipase A_2_-like (PLA_2_-like) toxins is a key step in their molecular mechanism, which involves oligomeric changes leading to the exposure of specific sites. Few studies have focused on the characterization of allosteric activators and the features that distinguish them from inhibitors. Herein, a comprehensive study with the BthTX-I toxin from *Bothrops jararacussu* venom bound or unbound to α-tocopherol (αT) was carried out. The oligomerization state of BthTX-I bound or unbound to αT in solution was studied and indicated that the toxin is predominantly monomeric but tends to oligomerize when complexed with αT. In silico molecular simulations showed the toxin presents higher conformational changes in the absence of αT, which suggests that it is important to stabilize the structure of the toxin. The transition between the two states (active/inactive) was also studied, showing that only the unbound BthTX-I system could migrate to the inactive state. In contrast, the presence of αT induces the toxin to leave the inactive state, guiding it towards the active state, with more regions exposed to the solvent, particularly its active site. Finally, the structural determinants necessary for a molecule to be an inhibitor or activator were analyzed in light of the obtained results.

## Introduction

Snakebite envenoming is considered a neglected tropical disease and represents one of the most severe public health problems in developing countries responsible for about 138,000 deaths per year^1^, causing more deaths and sequelae than well-known neglected tropical diseases, such as dengue hemorrhagic fever, cholera or Chagas disease, for example^2^. It is estimated that for 43% of snake species there is no specific therapy, which could be explained by the lack of research for the development of specific drugs or by the lack of production of specific anti-venoms for the treatment of victims from Africa, Asia and Latin America^3^. In addition, in several cases, the current serum therapy available shows limiting efficacy, justifying the importance of developing knowledge on this medical problem^4^.

Venomous snake species present a myriad of distinct toxins as neurotoxins and myotoxins, leading to paralysis, asphyxia, and death due to the damage of the neuromuscular transmission at the pre or postsynaptic sites, and pronounced muscle necrosis, respectively^5^. Many important myotoxins found in viperid snakes compose a subgroup of phospholipases A_2_ (PLA_2_s) referred as Lys49 myotoxins, Lys49-PLA_2_ homologues or PLA_2_-like myotoxins, whose components retain the phospholipase A_2_ scaffold but are not catalytic due to specific mutations, such as Asp49 → Lys and Tyr48 → Asn (system numbering from Renetseder et al*.* ^6–10^. In general, PLA_2_-like myotoxins present a local action^11^ and a significant number of other in vitro and in vivo activities, including membrane-destabilizing activity, inflammatory response (directly or indirectly induced by muscle necrosis), and inhibition effects, resulting from its affinity for biological molecules as heparin and coagulation factor Xa^12^. In addition, in vitro experiments have shown the ability of these myotoxins to affect different human cells, bacteria, fungi and parasitic organisms, thus showing that these snake toxins are potential candidates for the development of drugs against pathogens^13^.

The molecular mechanism of these myotoxins remains not yet fully understood, preventing the development of an effective antivenom serum therapy, although recent results based on experimental and theoretical works have shown progress on this area^14^. It is known that PLA_2_-like proteins have a homodimeric assembly which is related to its biological activity^15^, but these myotoxins can present very different quaternary arrangements^14,16–20^. The homodimeric conformation of PLA_2_-like proteins was further evidenced by the binding of α-tocopherol (αT; vitamin E) to these proteins, which could promote allosteric activation by the dimeric stabilization of the myotoxin BthTX-I from *Bothrops jararacussu*^17^. This finding describes the alignment of the C-terminal segments containing certain basic amino acid residues previously identified as essential for myotoxic activity^21–25^. Subsequently, Fernandes and colleagues^18^ proposed two regions: (i) MDoS—Membrane Docking Site and (ii) MDiS—Membrane Disruption Site, which could play a key role in the loss of membrane integrity. According to these authors, the two sites are aligned due to the quaternary conformation resulting from the binding of the allosteric activator, thus leading to myotoxin-membrane docking (MDoS interaction), followed by penetration (MDiS action) on the membrane, promoting its leakage and cell death. A more recent work^26^ has also suggested that the quaternary alignment and activation of the PLA_2_-like myotoxins can be triggered by the entrance of fatty acids in the hydrophobic channels of the myotoxin. In another work^27^, it was proposed a geometric description based on Euler's dimeric angles (roll, twist and tilt angles) of crystallographic structures of putative activated (bound to ligands) or inactivated (not bound to ligands) PLA_2_-like proteins, and suggested a transition mechanism between the active and inactive states, being modulated by the binding of an allosteric activator.

Mukherjee et al*.*^28^ reported that the αT ingestion is able to protect rats from lysosomal membrane disruption action of venom phospholipases A_2_ from Russell’s viper (*Daboia russelii*). According these authors, the polar interaction between the αT phenolic head group and the phosphate group of the lysosomal phospholipids could be a cause of this αT protective effect. These authors also mentioned that the release of free fatty acids and lysolecithin products arising from phospholipid hydrolysis can disrupt biological membranes^22^. Consequently, the higher αT plasma concentration of rats fed with αT could also increase the formation of complexes of this molecule with free fatty acids and lysolecithins and therefore to inhibit the lytic venom phospholipases activity against lysosomes^23^. Furthermore, Chandra et al*.*^29^ proposed a structural basis for the αT inhibition action on a venom catalytic phospholipase A_2_ also isolated from Russell´s viper, unlike structural studies of PLA_2_-like proteins and αT^17^ which proposed an activation role for this molecule.

Thus, in order to better understand the role of αT and similar molecules for PLA_2_-like proteins, we present here a comprehensive study with *apo-*BthTX-I and its complex with αT. In this work, we used bioinformatics techniques, including normal modes (NM) analysis, molecular dynamics (MD) simulations, and a hybrid NM/MD method combined with biophysical experiments (dynamic light scattering (DLS) and small-angle X-ray scattering (SAXS)) to achieve this goal.

## Results

### Biophysical experiments

In this work, the biophysical studies were carried out using dynamic light scattering (DLS) and small angle X-ray scattering (SAXS) methods, in order to observe several properties of the samples in solution, including size and molecular mass, dispersion and shape.

Dynamic light scattering (DLS) measurements of native BhTX-I in a basic environment (ammonium bicarbonate buffer—50 mM, pH 8.0) resulted an monomodal hydrodynamic radius (R_H_) of 2.0 nm with polydispersity (PD) of 15.7%. This R_H_ value is compatible with the dimeric assembly for PLA_2-_like myotoxins studied previously^9,19,30,31^, but the presence of monomers in this condition should not be ruled out, according to the observed PD values^32^. These assays indicate that under acidic conditions the toxin is found in a monomeric state^33^.

The addition and pre-incubation of αT with BthTX-I (basic environment) increased the mean R_H_ values of the samples in the DLS measurements for all BthTX-I:αT ratios evaluated (Table 1), particularly for higher concentrations of αT. In addition, mean PD values increased significantly compared to DLS measurements of *apo*-BthTX-I samples (Table 1), indicating a heterogeneous molecular size distribution for BthTX-I/αT complex samples.

**Table 1:** DLS measurements of *apo*-BthTX-I and BthTX-I/αT samples in an ammonium bicarbonate buffer (50 mm, pH 8.0) at 25 °C and final protein concentration of 2.5 mg/mL.

Sample	Hydrodynamic radius (mm)	Polydispersity (%)
Ap0-BthTx-I	2	15.7
BthTx-I/αT (1:0:5 ratio)	2.5	28.9
BthTx-I/αT (1:1 ratio)	3.5	26.3
BthTx-I/αT (1:10 ratio)	6.5	22.1

**Figure 1:**
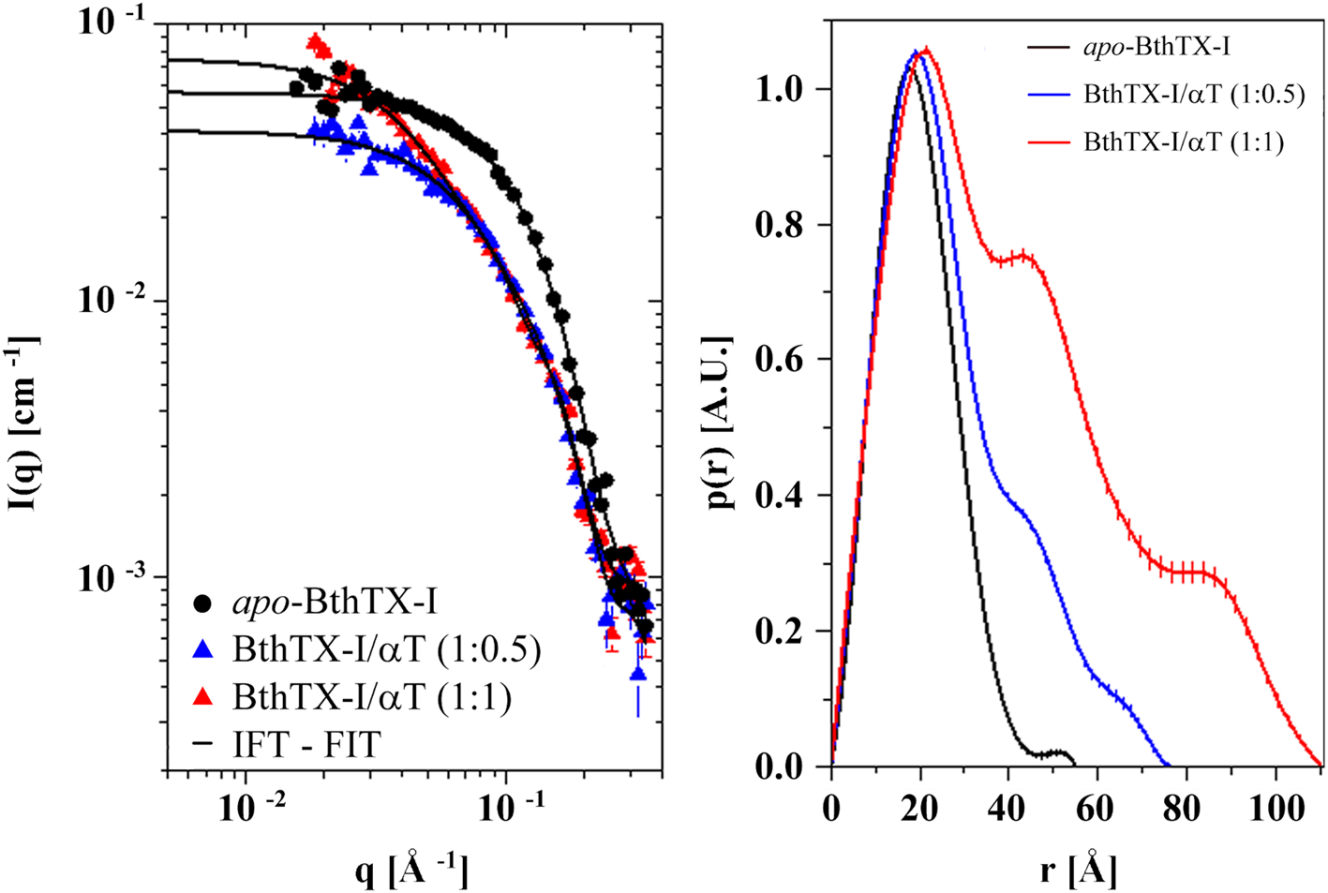
SAXS results for BthTX-I protein. Left panel: experimental SAXS profile to *apo*-BthTX-I (black symbols) and BthTX-I/αT complex with a molar ratio of 1:0.5 (blue symbols) and 1:1 (red symbols) described by IFT method. Right panel: pair distances distribution function p(r) of particles.

Throughout the acquisition of SAXS data, the samples were stable without any change in the scattering profiles for the studied samples. However, there were clear differences in the scattering intensities for *apo*-BthTX-I and pre-incubated samples with αT (Fig. 1). Indirect Fourier Transformation (IFT) analysis was used to describe the experimental SAXS profile, with the calculation of pair distances distribution function (p(r)) for each sample (Fig. 1). The structural parameters, radius of gyration (R_g_), maximum dimension (D_Max_) were also obtained (Supplementary Table S1). The p(r) function of isolated BthTX-I indicated a compact entity with a pair of distance within the particle with maximum probability at approximately 17.6 Å and a maximum diameter of ≈ 56 Å. In contrast, the p(r) function has a maximum diameter of ≈ 76 Å for the BthTX-I sample in the presence of αT in a molar ratio 1:0.5, with a maximum at 19 Å and two shoulders at ≈ 45 Å and ≈ 67 Å. With a 1:1 molar ratio, the p(r) profile extended to larger r values with a maximum diameter of ≈ 111 Å (approximately the double of *apo*-BthTX-I size), with the first maximum and shoulder at positions similar to αT in a 1:0.5 molar ratio, and a second shoulder at ≈ 87 Å. The p(r) functions indicate a clear change in the structural arrangement of BthTX-I when αT is present in solution^34^.

With a deeper understanding of the oligomeric states in the system, advanced modeling approaches were tried, assuming a mixture of different BthTX-I arrangements in the system. Thus, it was possible to observe the induction of BthTX-I oligomerization by the gradual formation of dimers (in particular at 1:0.5 molar ratio) and of tetramers (in particular at molar ratio 1:1) (Table 2 and Supplementary Fig. S1). Analysis using the OLIGOMER program^35^ shows a good agreement with experimental data in the case of *apo*-BthTX-I and in the presence of αT in molar ratio 1:0.5; however it is clear that in the case of 1:1 in low q values range, the fit is not able to describe the experimental data. This indicates that one might have larger structures in the system. In order to investigate this hypothesis, Debye analysis was applied to the sample (more details in Methods section). The analysis, assuming a linear arrangement of the BthTX-I monomers in the presence of αT, describes the experimental SAXS data fairly well (Table 3 and Supplementary Fig. S2).

**Table 2:** Oligomeric fractions of *apo*-BthTX-I and BthTX-I/αT samples in ammonium bicarbonate buffer (50 mm, pH 8.0) at 25 °C and analyzed by their SAXS data and their crystallographic structures.

Sample	OLIGOMER program volume fraction		
	Monomers	Dimers	Tetramers
*apo*-BthTx-I	100	–	–
BthTx-I/αT (1:0:5 ratio)	71	29.1	–
BthTx-I/αT (1:1 ratio)	67	11	32

**Table 3:** Oligomeric fractions of BthTX-I/αT samples in ammonium bicarbonate buffer (50 mm, pH 8.0) at 25 °C and analyzed by their SAXS data using the Debye approach of linear arrangements of the monomeric crystal structure.

Sample	OLIGOMER program volume fraction			
	Monomers	Dimers	Tetramers	Dij (Å)
BthTx-I/αT (1:0:5 ratio)	77	8	15	47 ± 1
BthTx-I/αT (1:1 ratio)	33.2	1.2	65.6	54 ± 4

Thus, despite solubilization in an alkaline buffer, the *apo-*BthTX-I sample presented a majority of monomers in this condition composition. At the same time, there was an equilibrium of monomers, dimers, and larger aggregates (as tetramers) in the samples pre-incubated with αT, with different distributions depending on the models used in the analysis (Tables 2 and 3, and Supplementary Figs. S1 and S2).

### Computational analyses

In this work, we carried out MD (Molecular Dynamics) and MDeNM (Molecular Dynamics with Excited Normal Modes)^36^ simulations to generate several structures of the dimeric *apo*-BthTX-I and BthTX-I/αT complex aiming at exploring the accessible conformational space by this PLA_2_-like myotoxin.

Initially, we selected the eight lowest frequency normal modes of the *apo*-BthTX-I and BthTX-I/αT systems according to their respective fluctuations (Supplementary Fig. S3) and their conformational movement for visual inspection. Then, these normal modes were combined, resulting in 54 and 48 vectors (or replicas) for the *apo*-BthTX-I and BthTX-I/αT complex, respectively. The two molecular systems were then kinetically driven 10 times in the direction of these vectors by successive energy input corresponding to a 3 K increase in the temperature of the entire system^36^, giving 540 and 480 structures, respectively, constituting structure set 1. These structures had a considerable similarity of secondary structures compared to the crystallographic structure of BthTX-I/αT determined by dos Santos and colleagues^17^: the experimental model had 41%/7% of α-helix/β-sheet content, while the *apo*-BthTX-I and BthTX-I/αT structures from set 1 showed, 38.8 ± 1.6%/7.1 ± 0.9% and 39.6 ± 1.4%/8.1 ± 0.9% of α-helix/β-sheet content, respectively (Table 4). Thus, the prototypical secondary structure of the PLA_2_-like proteins^8^, which includes an N-terminal α-helix (Helix-I), a putative Ca^2+^-binding loop, two antiparallel α-helices (Helix-II and Helix-III), two antiparallel β-sheets (β1 and β2) and the C-terminal loop segment, were preserved. The tertiary structure of each subunit in the *apo*-BthTX-I and BthTX-I/αT systems was also maintained because their average backbone RMSD values with respect to the respective starting structures were always less than 1.0 Å (Table 4).

**Table 4:** Backbone RMSD and secondary structure percentage for *apo*-BthTX-I and BthTX-I/αT of structures from set 1.

	*apo*-BthTx-I	BthTx-I
	RMSD (Å)	
Monomer A	0.78 ± 0.14	083 ± 0.09
Monomer B	0.81 ± 0.09	0.78.0.08
	Secondary structure (%)	
Α-Helix	38.76 ± 1.62	39.62 ± 1.44
Β-Sheet	7.10 ± 0.93	8.10 ± 0.91

**Figure 2:**
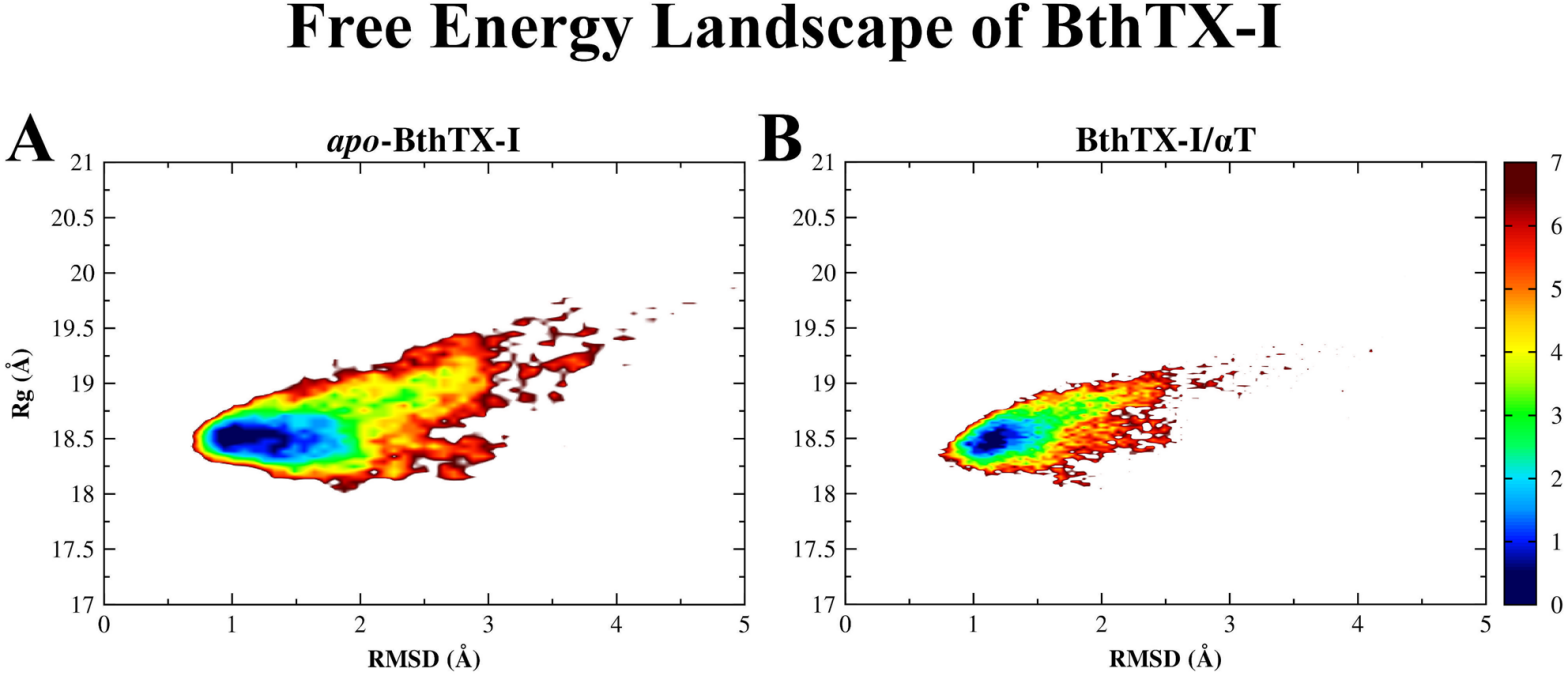
Free energy landscape (kJ/mol) of the *apo*-BthTX-I (**A**) and BthTX-I/αT complex (**B**) systems using RMSD and radius of gyration (R_g_) as reaction coordinates. The most and least visited conformations are located in a range of colors from blue to red, respectively, as indicated on the left palette. Set 1 structures were submitted to a further step of 50 ps of free-MD, collecting frames every 1 ps.

**Figure 3:**
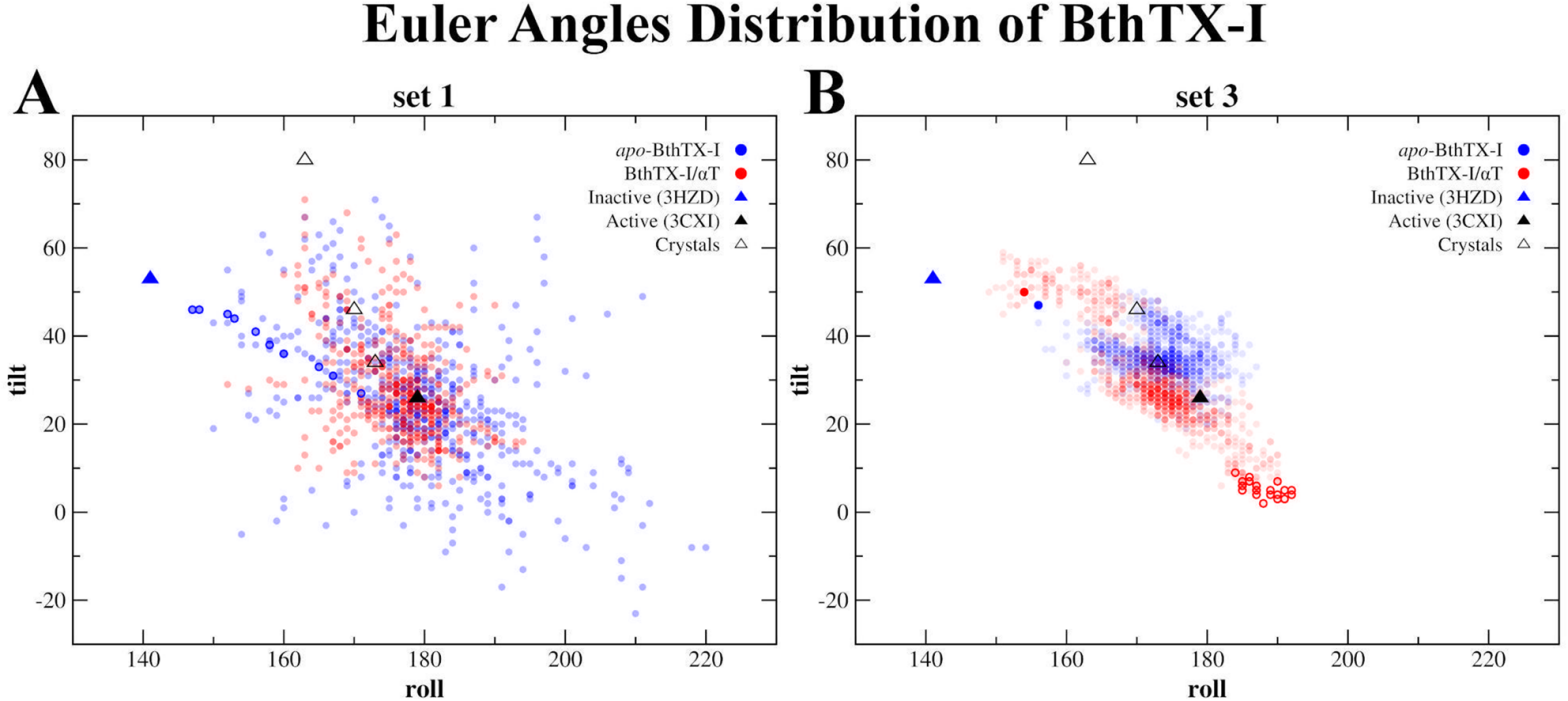
Distribution of the Euler roll/tilt angles of BthTX-I from structure set 1 (**A**) and structure set 3 (**B**). The structures of *apo*-BthTX-I (blue circles) and BthTX-I/αT complex (red circles) are represented as transparent filled circles. The inactive (PDB id 3HZD) and active (PDB id 3CXI) states are displayed in solid triangles in blue and black, respectively, while the other active structures are presented as unfilled triangles, extracted from the following PDB ids: 1QLL (roll/tilt: 163°/80°), 1XXS (roll/tilt: 170°/46°) and 3QNL (roll/tilt: 173°/34°). In structure set 3 (**B**), the starting structure of each system is represented by solid circles in blue (*apo*-BthTX-I) and in red (BthTX-I/αT complex). Some structures of the BthTX-I/αT system with lower tilt values are represented by red contoured circles.

In contrast, the quaternary conformations of the *apo*-BthTX-I and BthTX-I/αT systems were diverse, as shown by the distribution of the Potential of Mean Force (PMF) using the RMSD and R_g_ values as reaction coordinates (Fig. 2). Regarding the backbone RMSD values of these structures, *apo-*BthTX-I showed a more diverse set of conformations, presenting an RMSD coverage area within the limits of 5 kcal/mol, while BthTX-I/αT covered a reduced area of ≈ 3 kcal/mol, suggesting a clear structural restriction induced by the αT molecules (Fig. 2). On the other hand, the slight differences between the radii of gyration of the *apo* and αT-complexed models in set 1 (20.16 ± 1.33 Å and 19.03 ± 0.64 Å for *apo*-BthTX-I and BthTX-I/αT, respectively) did not reflect the diversity observed in RMSD calculations. In agreement with the RMSD results, the distribution of the Euler angles (roll/tilt angles) of BthTX-I showed the same trend, with BthTX-I/αT exploring less of the Euler angles space, especially around the roll/tilt region of 200°/ − 10°, with roll values ranging from 152° to 194° and tilt from 6° to 71°, while *apo-*BthTX-I showed roll values from 147° to 220° and tilt from − 23° to 71° (Fig. 3A). According to Borges et al.^27^, roll/tilt Euler angles are valuable quantities for characterizing active and inactive states and, for this reason, they were used to determine the conditions that BthTX-I visits these states.

The broader exploration of roll/tilt angles observed in the *apo-*BthTX-I system in set 1 resulted in some NM combination vectors capable of inducing displacement of BthTX-I towards the inactive state (PDB id 3HZD, roll/tilt values of 141°/53°)^27^, suggesting that αT could constrain BthTX-I to remain in the active state (Fig. 3A). Therefore, we carried out further MDeNM simulations using the combined NM vector, which best led the *apo-*BthTX-I system towards the inactive state (Fig. 3A, dark-blue circles), which we applied to both systems in order to verify the effect of αT in this transition. Thus, 200 new structures (10 replicas but keeping the same NM combination vector with 20 excitation steps) were generated, which were grouped into a new set defined as set 2. Surprisingly, these new simulations showed that the excitation of the two *apo-*BthTX-I and BthTX-I/αT along the selected vector could propagate structures with roll/tilt angles values similar to other inactive PLA_2_-like proteins^27^ (Supplementary Fig. S4), thus, indicating that αT cannot prevent the active-to-inactive state transition when it is forced.

In addition, the replica of each *apo-*BthTX-I and BthTX-I/αT system of set 2 with the roll/tilt angle values closest to those of the inactive state (Supplementary Fig. S4, strong colored lines) were subjected to 50 ps supplementary free MD simulations in order to eliminate the excess kinetic energy. The resulting structures were carefully analyzed to exclude those presenting a dimer disruption caused by the excitation exhaustiveness by examining their overall RMSD values for both *apo*-BthTX-I and BthTX-I/αT. Therefore, the structures corresponding to the putative inactive state of each system were selected based on the lowest backbone RMSD value with respect to the inactive *apo*-BthTX-I crystallographic structure elucidated by Fernandes and colleagues^9^ (PDB id 3HZD). For the *apo-*BthTX-I system, the closest structure to the inactive state had roll/tilt angle values of 156°/47° and a backbone RMSD of 2.92 Å compared to the inactive BthTX-I^9^, while these values were 154°/50° and 2.66 Å for the corresponding structure in the BthTX-I/αT system. To verify their structural stability these two forms of *apo-*BthTX-I and BthTX-I/αT were submitted to an additional 10 ns free-MD simulation, resulting in 1000 structures for each system, that were grouped in set 3.

**Figure 4:**
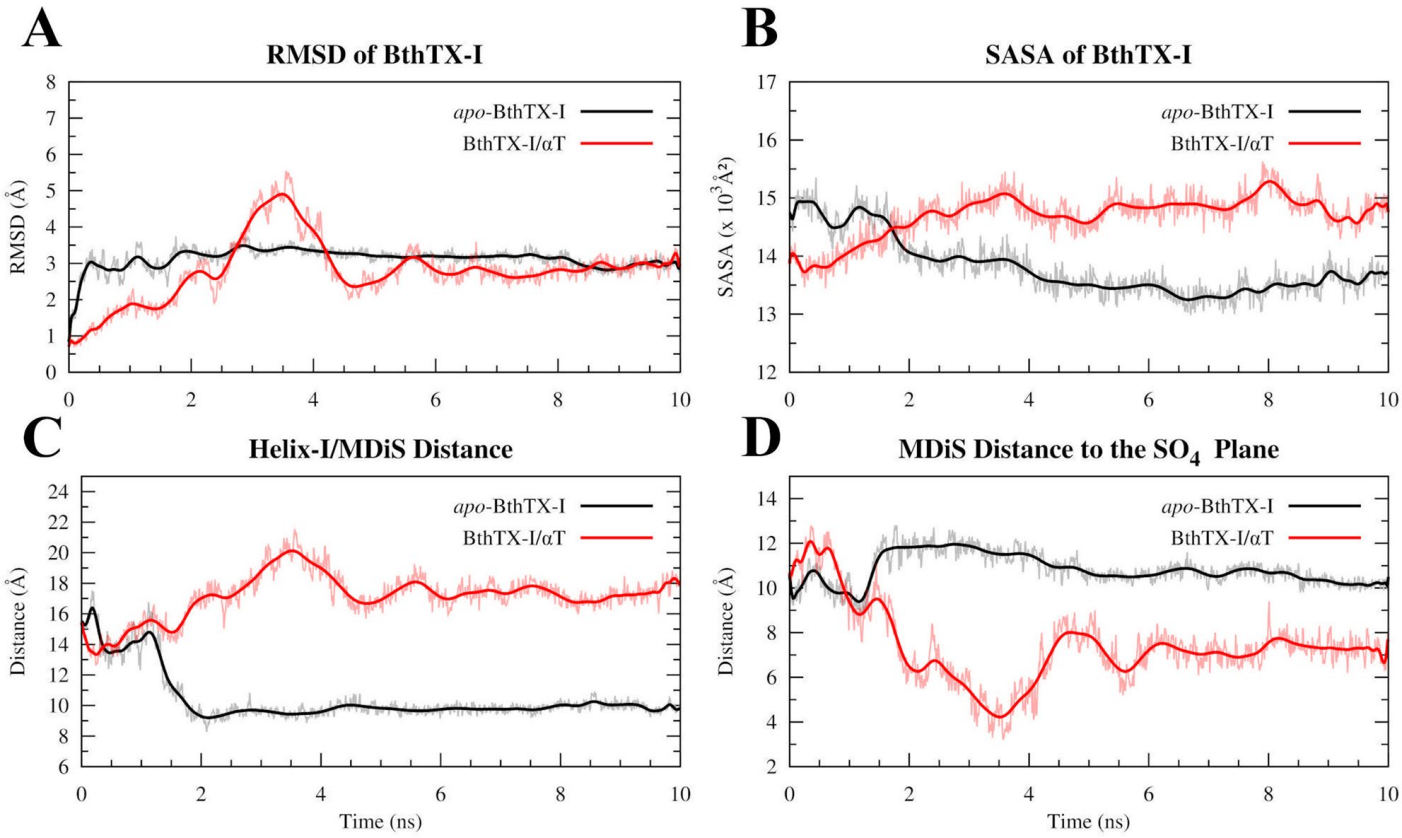
Structural analysis of the structure set 3 for *apo*-BthTX-I (black lines) and BthTX-I/αT complex (red lines) systems, considering the following variables: (**A**) RMSD, (**B**) SASA, (**C**) Helix-MDiS, and (**D**) MDiS distance to sulfate plane (SO_4_ plane).

The structures of set 3 had average roll/tilt angles of 171.92° ± 7.75°/35.23° ± 4.74° and 173.64° ± 4.70°/29.12° ± 11.50° for the *apo*-BthTX-I and BthTX-I/αT systems, respectively (Fig. 3B). These mean roll/tilt angles are compatible with the active conformations cited by Borges et al.^27^ (roll angles > 160° and tilt angles generally < 50°), indicating that an inactive BthTX-I conformation can follow a path towards the active state, whether or not bound to αT molecules. Moreover, it was possible to note that BthTX-I/αT showed a more dispersed angle distribution, reflecting a better occupation of the Euler angles space (Fig. 3B), which is in agreement with the backbone RMSD calculations for this system, although both *apo*- and αT-complexed BthTX-I converged to a backbone RMSD value around 3 Å (Fig. 4A). Despite this, the structures of set 3 for the *apo*-BthTX-I and BthTX-I/αT systems have other remarkable structural differences when analyzed in detail. Particularly with regard to the following reaction coordinates: the distance between the Helix-I and MDiS regions, solvent-accessible surface area (SASA) and the distance of both MDiS regions to a sulfate plane. This plane was previously described by studies with a PLA_2_^37^ and a PLA_2_-like protein^17^, which suggested that sulfate ions form a plane in PLA_2_ dimeric X-ray structures and mimic the phosphate head groups of membrane phospholipids. For PLA_2_-like structures, these sulfate ions interact with a cluster of basic residues on the C-terminal region (MDoS) mimicking the phosphate head groups of the phospholipid membranes.

The residues of the BthTX-I/αT were more exposed to the solvent, as evidenced by the SASA values (Fig. 4B), and the Helix-I/MDiS distance was around 18 Å, while in *apo*-BthTX-I it was much shorter, between 10 and 12 Å (Fig. 4C). Also, it is worth note that MDiS regions were able to approximate the sulfate plane in the BthTX-I/αT system, reaching values less than 8 Å (Fig. 4D, red line). On the other hand, *apo*-BthTX-I showed a tendency to maintain its MDiS region at around 11 Å from the sulfate plane during almost the entire MD simulation (Fig. 4D, black line).

**Figure 5:**
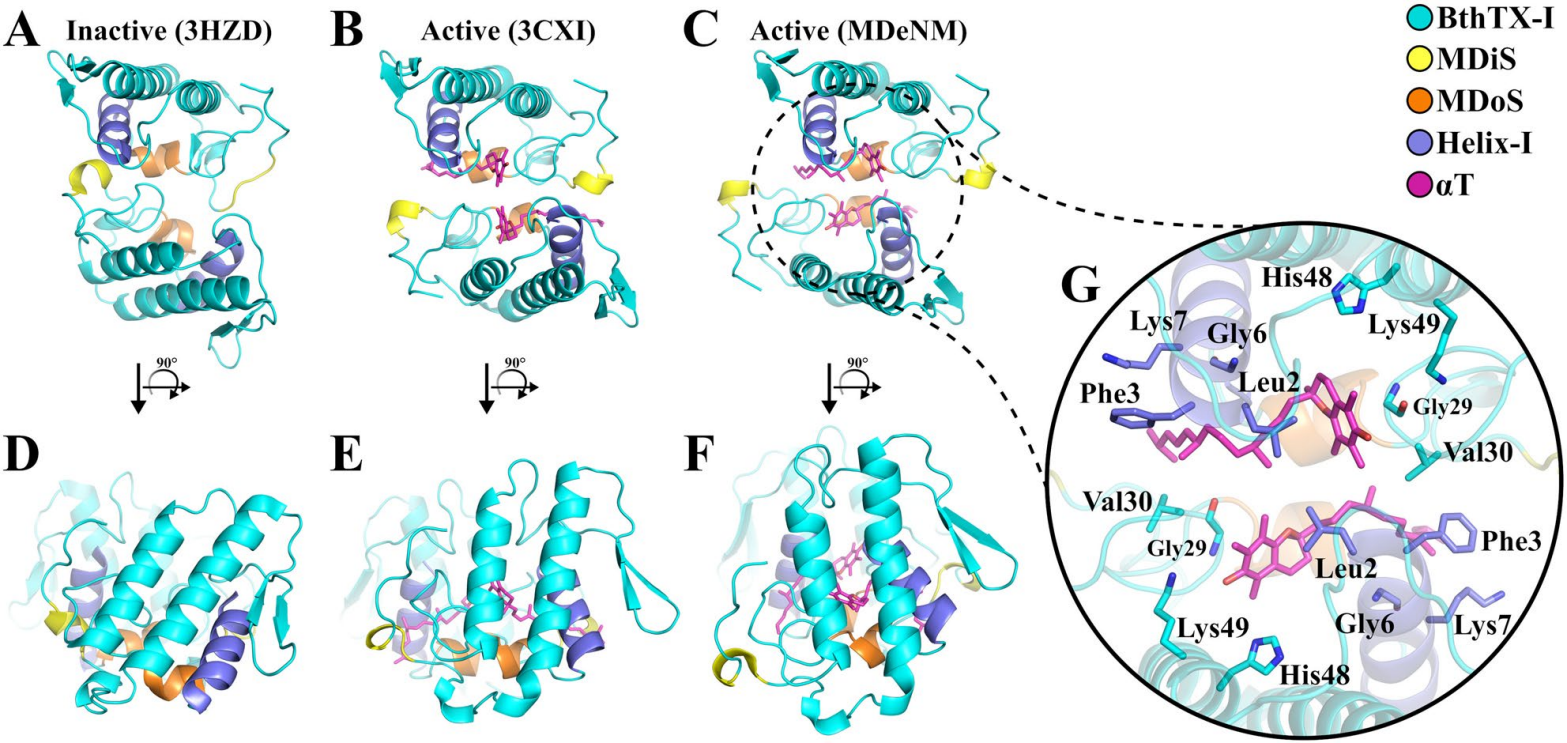
Top-down view of (**A**) the inactive (PDB id 3HZD, roll/tilt: 141°/53°) and (**B**) active (PDB id 3CXI, roll/tilt: 179°/26°) states, and (**C**) a representative structure of set 3 (roll/tilt: 189°/5°) and their respective side views (rotation of 90°) shown in (**D**,**E**,**F**), presenting the different dimeric orientations in each state. (**G**) The active state of BthTX-I is induced by two αT molecules (magenta), and the BthTX-I side-chain residues that most interacted with αT in MD and MDeNM simulations are shown as stick and colored according to its respective positions in the protein. The structurally important regions of BthTX-I, as the MDiS and MDoS domains, and Helix-I are represented in yellow, orange and purple, respectively.

The structures of set 3 showed conformational differences in comparison to the inactive and active states (Fig. 5). Thus, to analyze in details the main interactions which stabilize the BthTX-I/αT complex, the prevalence of contacts between the BthTX-I residues and αT were calculated for the structures of sets 1 and 3 (Supplementary Table S2). For these two sets, some residues of Helix-I (Leu2, Phe3, Gly6 and, Lys7) and of the calcium-binding loop (Gly29 and Val30) remained in contact with αT for almost 100% of the structures. Furthermore, some hydrophobic channel residues (His48 and Lys49) also presented relevant contacts with αT in more than 60% of the structures (Fig. 5G and Supplementary Table S2). On the other hand, the MDiS residues Leu111 and Phe114 showed a lower prevalence of contacts with the ligand, particularly in set 3, when active conformations were reached after a 10 ns MD simulation was performed with the selected inactive model of the BthTX-I/αT.

## Discussion

### The oligomerizing property of PLA_2_-like myotoxins

BthTX-I is a model protein in the group of PLA_2_-like proteins, which are mainly called myotoxins because of their myotoxic effect and other toxic effects by their membrane destabilization activity^14,23,38^. It has been shown that these proteins may assume a dimeric conformation when in solution^9,18^; in other cases, their degree of oligomerization is concentration-dependent^39^, or related to the binding of particular ligands^20,40^. Furthermore, it is important to emphasize that their activity on the membrane is dependent on dimeric conformation^15,41^. The current proposal suggests that changes in dimeric interface are necessary to allow these proteins to convert between the active and inactive states modulated by an allosteric activator^14,26^. Therefore, to better understand the oligomeric aspects of BthTX-I, we used biophysical experiments associated with in silico simulations.

DLS measurements of the *apo*-BthTX-I sample present an average R_H_ of 2.0 nm with a PD of 15.7%, suggesting the presence of dimers in solution, as have been observed with this and other PLA_2_-like myotoxins^19,30,39,42,43^. Since the theoretical R_H_ values between the BthTX-I monomers and dimers are close, in its coverage of dispersion value (PD), it was understood that the sample also presents monomers in solution (Table 1). This hypothesis was corroborated by SAXS measurements of the *apo*-BthTX-I sample, which showed a majority of monomers (Tables 2 and 3 and Supplementary Figs. S1 and S2). This unexpected oligomeric assembly for a PLA_2_-like myotoxin^26,30,42^ may be the result of its isolation from snake venom, which has been performed using abrasive solvents and extremely acidic solutions (reverse phase chromatography), allowing the dissociation of the dimeric structure^33,44^. On the other hand, pre-incubation with αT indicated the increase of mean R_H_ and PD of BthTX-I/αT samples, suggesting a heterogeneous molecular size distribution and/or the formation of more extended elongated oligomeric assemblies (Tables 2 and 3). SAXS measurements of these samples showed an equilibrium between monomers, dimers and larger aggregates (Fig. 1 and Supplementary Figs. S2 and S3). Thus, there was the gradual formation of dimers (in particular at a 1:0.5 molar ratio), tetramers (at a 1:1 molar ratio) (Tables 2 and 3, Supplementary Figs. S1 and S2) and larger aggregates (at 1:10 molar ratio) (Table 1) by increasing the molar proportion of αT, demonstrating its capacity to promote the oligomerization of PLA_2_-like toxins.

In order to study the conformational changes of BthTX-I at the atomic level, MDeNM simulations were performed for *apo*-BthTX-I and BthTX-I/αT systems, generating many structures, corresponding to set 1. These structures were essential to describe the conformational space of BthTX-I, which showed that the toxin exhibits higher conformational changes when αT is not bound (Fig. 2), suggesting that αT is important for stabilizing the structure of the toxin. Indeed, the biophysical results obtained here also showed the importance of the complexation between the toxin and αT to stabilize its oligomeric conformation (Tables 1, 2, and 3). The Euler angles distribution^27^ is also in agreement with the results obtained here, which showed that the *apo*-BthTX-I had a larger dispersion of angles in the absence of αT (Fig. 3A). Although a large set of conformations were generated for the two systems, the tertiary structure of the toxin was preserved. This was shown by the low RMSD values and the similar secondary structure percentage, with respect to the starting structure (Table 4), which indicates an appropriate exploration of quaternary conformational changes.

The roll/tilt angles distribution is a valuable measure to determine the corresponding structures in the active and inactive states, as previously reported by Borges et al.^27^. Therefore, we analyzed the set 1 structures according to these two angles to describe the transition from the active to inactive state. A significant result of the transition between these states was that only the *apo*-BthTX-I system could reach the inactive state (Fig. 3A), suggesting that the αT molecules could stabilize BthTX-I in the active state.

### αT maintains BthTX-I in the active state

Previous studies^14,26^ have classified the crystallographic structures of many PLA_2_-like proteins and have shown that the active state is reached when complexed with an allosteric activator, such as fatty acids^45–47^, αT^17^ and even PEG molecules^9,18,39^. On the other hand, when these proteins are found in the absence of any ligand, the inactive state is observed^9,18^. Therefore, would it be possible for BthTX-I to reach a stable conformation in the inactive state when it is bound to αT molecules? To address this question, we performed additional MDeNM simulations by exciting the two systems (*apo*-BthTX-I and BthTX-I/αT) using combinations of NM vectors that lead to the inactive state and further MD simulations to determine the stability of the structures. Both systems were subjected to these MDeNM simulations using only combined NM vectors pointing towards the inactive state originating from the prior calculations on the *apo*-BthTX-I system, as shown in Fig. 3A (dark-blue circles), the generated structures constituted set 2. As observed in the Euler angles distribution (Supplementary Fig. S4), both systems were able to reach the inactive state when a guiding kinetic impulsion was applied. However, we also studied these inactive structures with longer free MD simulations in order to avoid any biased result; these structures constituted set 3. Accordingly, the Euler angle analysis of the set 3 structures showed a tendency to return to the active state, as observed by the decrease of tilt angles (Fig. 3B). However, the presence of αT induced BthTX-I to reach lower tilt angle values, with certain structures showing values below 20°. Indeed, structural analysis of Lys49-PLA_2_-like toxins crystallographic structures defined the dimer orientation relative to this angle as a relevant structural characteristic that differs the active and inactive states, and such decrease is strongly associated with the activation process of these toxins^27^.

Furthermore, αT strongly interacts with Helix-I of BthTX-I in sets 1 and 3 (Supplementary Table S2), as observed by the contact prevalence analysis, which suggests that this region plays a role in the structural stabilization of the toxin and the allosteric activation. Indeed, the importance of Helix-I for PLA_2_-like toxins has been revealed by previous studies on the cleavage of Helix-I, reducing the activity of these toxins by approximately 75%^13^. In addition, the binding of rosmarinic acid to this region results in approximately 90% inhibition of the toxin^48^. In contrast, the interaction between αT and MDiS residues such as Leu111 and Phe114 was diminished when the toxin reaches the active state (Supplementary Table S2), indicating that this region could be more accessible to the solvent. In agreement with this finding, the accessible surface area of BthTX-I is higher in the presence of αT (Fig. 4B). The MDiS is a major region of PLA_2_-like toxins related with their activity^18^, supported by mutagenesis^22,25,49^, synthetic peptides^12,21^ and inhibitory experiments^19,30,42,50^.

Taken together, the decrease in tilt angle, the increase in the accessible surface area and the exposure of MDiS for BthTX-I, suggest that αT can force the toxin to leave the inactive state, guiding it to the active state, with more regions exposed to the solvent. Therefore, these structural aspects show, for the first time, the ability of αT to induce BthTX-I to visit preferentially the active state conformations observed in the present work, reinforcing the importance of allosteric activators and the current proposal concerning the structural events that lead to the myotoxic activity^18,26^.

### The allosteric activator effect on BthTX-I

Five essential steps can describe the myotoxic mechanism for PLA_2_-like toxins: (i) entrance of a hydrophobic molecule into the hydrophobic channel of the protein; (ii) opening of the secondary hydrophobic channel; (iii) entrance of a second hydrophobic molecule into the secondary hydrophobic channel; (iv) conformational change of the dimer leading to an alignment of the MDoS (Membrane Docking Site) and exposure of the MDiS and (v) interaction of the MDiS with the cellular membrane. The last step leads to membrane leakage and cell death^14,27^. In the present work, we showed how the active state can assume different conformations, leading to exposure of the MDiS (Fig. 4C) and the approximation of the MDoS region or sulfate plane (Fig. 4D).

Indeed, studies of the molecular mechanisms for the inhibition of PLA_2_-like toxins with different molecules have confirmed the proposal of myotoxic sites. It has been observed that these molecules inhibit PLA_2_-like proteins in different ways, including: (i) binding to Helix-I/MDiS region, physically blocking access to the hydrophobic channel (e.g., rosmarinic^48,50^, chicoric^30^, and aristolochic acids^4^); (ii) binding to the MDoS (e.g., caffeic acid^4^ and suramin^20^); (iii) binding in the hydrophobic channel (e.g., caftaric acid^42^, p-bromophenacyl bromide (BPB)^9^; Varespladib^51^ and zinc ions^26^); (iv) binding to the MDiS (e.g., aristolochic^4^, chicoric acid^30^, zinc ions^26^, suramin^19,20^ and Varespladib^51^); or (v) induction of the toxin’s oligomerization (e.g., suramin^19^). Although the binding regions of the inhibitors are well described, the structural importance of an allosteric activator is only related to the dimer reorientation^14,26^. Remarkably, no study has yet described how an allosteric activator can lead these proteins to express their myotoxic activity and, more importantly, what characteristics distinguish it from an inhibitor.

**Figure 6:**
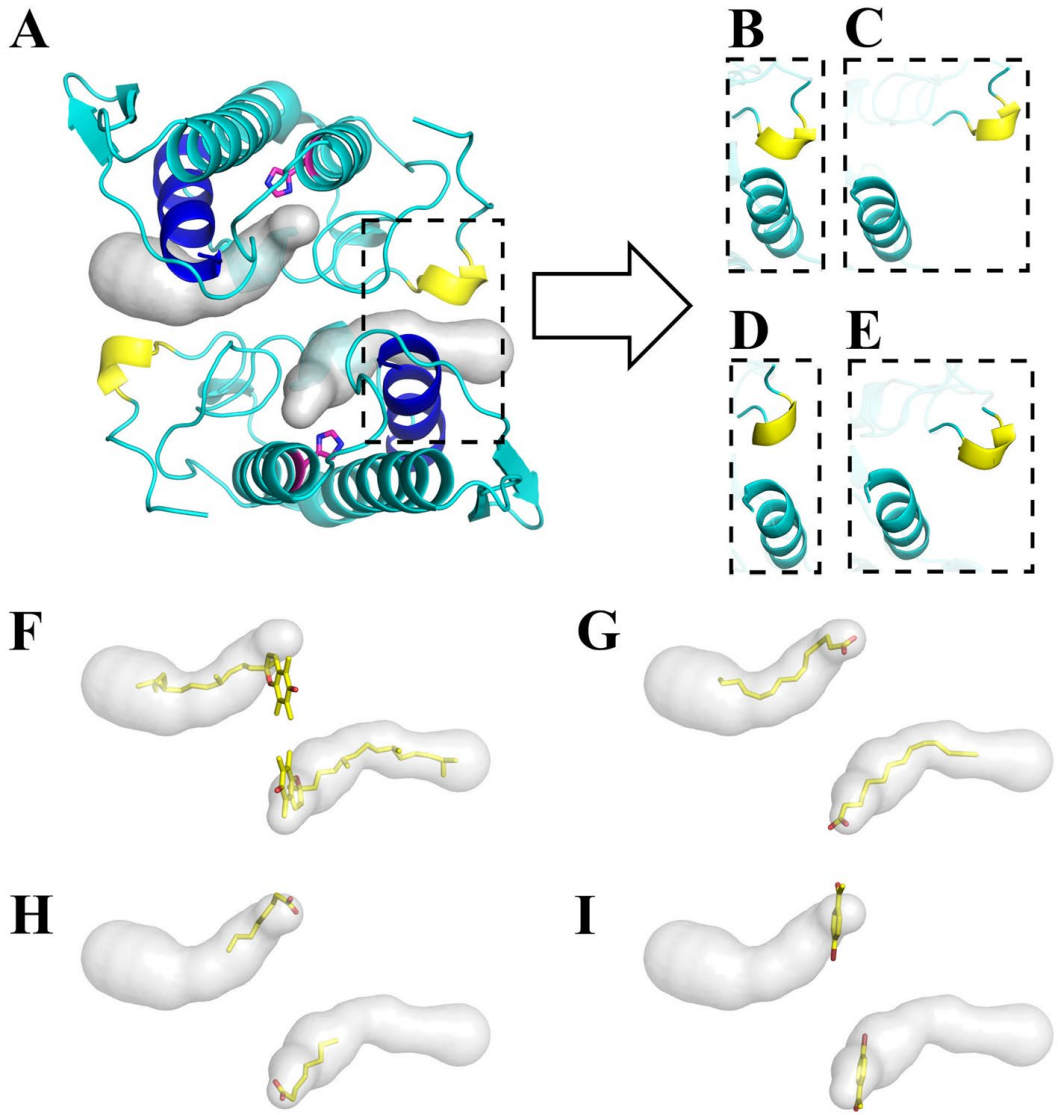
Structural aspects of the BthTX-I. (**A**) The structure of the dimeric BthTX-I is represented in cyan, with Helix-I and MDiS highlighted as blue and yellow, respectively. His48 residues of each subunit are represented in magenta, indicating the entrance of the hydrophobic pocket, which extends to the Helix-I/MDiS region (represented as a gray surface). Helix-I and MDiS regions present structural dynamics with the approximation or separation movement to the inactive or active state. (**B**) The inactive configuration obtained by MDeNM simulations taken from structure set 2 presents a short Helix-I/MDiS distance. (**C)** Same as in B but after 10 ns of free MD (structure set 3). These regions are separated, with MDiS exposed to the solvent. (**D**) The same region corresponding to the crystallographic structure in the active state (PDB id 3CXI) shows similar behavior (**E**) after 100 ns of free-MD. It can be observed that the Helix-I/MDiS distance is modified by the presence of αT during the activation process. A detailed view of the hydrophobic pocket being occupied by different molecules, which can act as allosteric activators: αT (**F**) and (**G**) long (PDB id 6B80); or inhibitors: short-chain fatty acids (**H**) (PDB id 6B83) and *p*-bromophenacyl bromide (PDB id 3HZW).

The dimeric structure of BthTX-I presents two hydrophobic channels occupied by αT molecules, whose entrances are flanked by Helix-I and MDiS, and are exposed to the protein surface in opposite sides, as shown in Fig. 6A. Previous studies with other PLA_2_-like proteins have shown that these channels can also be occupied by fatty acids or PEG molecules^18,45–47^, suggesting a structural and functional relevance to this region (Fig. 6F–I). In this sense, we analyzed the distance between Helix-I and MDiS in set 3, since they form the entry of the hydrophobic channels. When αT is not present, the MDiS and Helix-I of BthTX-I tend to approach each other, assuming a stable conformation with the two hydrophobic channels closed to the solvent (Fig. 4C, black line). On the other hand, when αT is present, the distance of these regions is increased (Fig. 4C, red line, and Fig. 6B,C). In support of these results, 100 ns MD simulations showed the same Helix-I/MDiS interaction scheme (Fig. 6D,E and Supplementary Fig. S5), suggesting that the allosteric activator disturbs the contact of the MDiS with Helix-I of the toxin, allowing the MDiS regions to move freely in the solvent.

**Figure 7:**
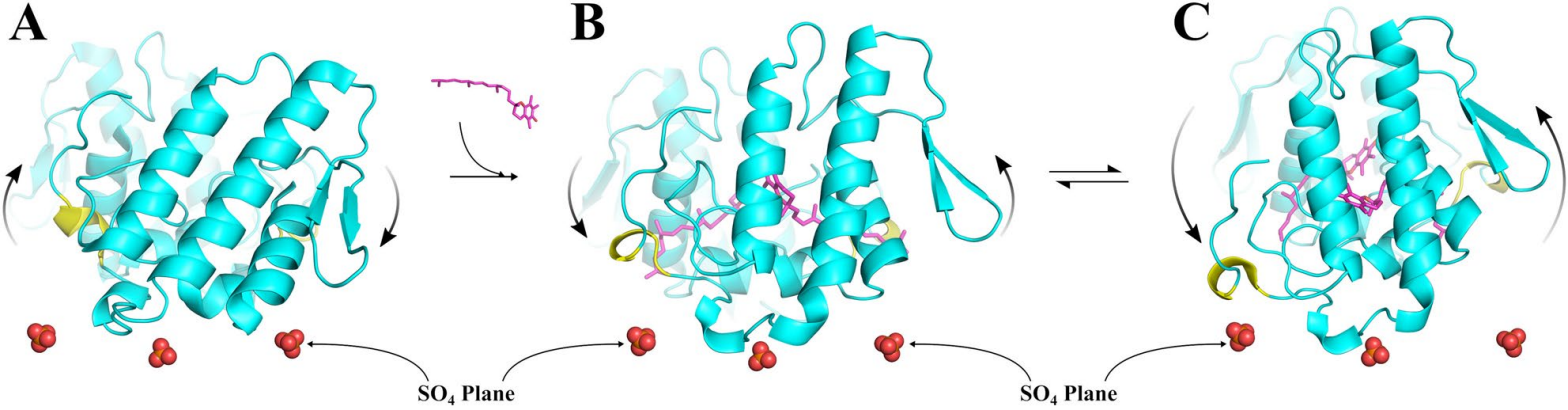
The allosteric activation mechanism of the BthTX-I induced by αT in respect of the sulfate plane, considered to mimic the cellular membrane^37^. (**A**) The inactive state keeps the MDiS regions (yellow) far from the sulfate plane and in contact of Helix-I. (**B**). The binding of an allosteric molecule (αT, represented in magenta) to BthTX-I (cyan) induces the protein to be reoriented, leading to MDiS exposure and approximation of the sulfate plane. (**C**) The active state shows a dynamical behavior, being able to assume greater MDiS exposure and closer approximation to the sulfate plane in comparison to crystallographic structures, increasing the chance to induce membrane leakage.

As αT induces BthTX-I to expose its MDiS to the solvent (Fig. 4C), this region could adopt different conformations, making motion to the membrane possible. To test this hypothesis, the mean distance of both MDiS regions of the toxin to the crystallographic sulfate plane was calculated (the sulfate plane is formed by the sulfate binding sites of the protein^37^ and it is related to its MDoS region^17^). As expected, the *apo*-BthTX-I system showed a higher distance to the sulfate plane, presenting values of 10–12 Å (Fig. 4D, black line), which could be related to the stabilization of the MDiS, due its interaction with Helix-I (Fig. 4D, red line). In contrast, when the interaction between Helix-I and MDiS is prevented by αT, the MDiS regions of the BthTX-I can move freely, approaching the sulfate plane, reaching lower distances of almost 4 Å (Fig. 4D). Interestingly, the distance to the sulfate plane and the tilt angle showed a strong correlation (R = 0.92), showing that reduced distances to this plane corresponds to lower tilt values (Fig. 3B, red circles with contour). Therefore, this movement can constitute the most important conformational change related to the activation of this group of toxins, allowing the interaction of MDiS with the membrane (Fig. 7 and Supplementary Video).

The current structural data available in the literature concerning PLA_2_-like toxins complexed with ligands do not explain the structural and physicochemical characteristics that distinguish an inhibitor from an activator. The present work shows that αT is composed of a long aliphatic chain (Fig. 6F), similarly to a fatty acid (Fig. 6G), which is capable of filling the hydrophobic channel and playing a central role as an allosteric activator for PLA_2_-like proteins, being essential for MDiS modulation as discussed above (Fig. 4C,D and 6B–E)..Indeed, a study with a PLA_2_-like toxin (MjTX-II, isolated from *B. moojeni* venom) bound to fatty acids of different sizes of hydrocarbon chain showed a higher conformational stability of the toxin after interaction with long-chain fatty acid molecules (Fig. 6F,G)^47^. However, when a small molecule binds to the hydrophobic channel and it only partially occupies this channel, the ligand can behave as an inhibitor, as observed for short fatty acids (Fig. 6H) and BPB (Fig. 6I)^9^. According to the present work, long-chain molecules can fill the hydrophobic channel disrupting the Helix-I/MDiS interaction, leading to exposure of the MDiS, allowing its interaction with the membrane. In contrast, small molecules are not able to disrupt the Helix-I/MDiS interaction and can prevent long chain molecules from binding into the hydrophobic channel, thus acting as inhibitors. In particular, BPB binds covalently to His48 of the hydrophobic channel and prevents the interaction of fatty acids or αT molecules^9^. Thus, as previously pointed out^47^, the length of a particular ligand is a fundamental characteristic and may explain why different molecules bound to the hydrophobic channel can act as an inhibitor or an allosteric activator.

## Concluding remarks

PLA_2_-like toxins are the main component of many venoms and play a key role in the local effects in the victims of ophidian accidents, which are inefficiently neutralized by serum therapy^52^. Therefore, researchers have sought effective inhibitors of these toxins in the past two–three decades; however, little attention has been paid to how allosteric activators modulate these proteins. The present work shows in details how these allosteric activators can modulate these toxins in order to favor their dimerization and to adopt active conformations, with high exposure of their MDiS regions, an essential molecular event related to the functional aspects of this protein. Above all, for the first time, it is discussed how different molecules (e.g. long or short chain molecules) that bind to the same binding site (hydrophobic channel) can act as allosteric activators or inhibitors. The understanding of all myotoxic mechanism details and, also structural features of potential activators and inhibitors are valuable knowledge that could be used to rational drug design to inhibit these snake venom toxins.

## Methods

### Biophysical experiments

#### Dynamic light scattering (DLS)

DLS measurements were performed with *apo*-BthTX-I and BthTX-I/αT complex samples using a DynaPro Titan device (Wyatt Technology) at room temperature (25 °C). BthTX-I and αT were solubilized with an ammonium bicarbonate buffer (50 mM, pH 8.0). Protein and ligand were pre-incubated for at least 10 min at 1:0.5, 1:1 and 1:10 molar ratios of BthTX-I:αT, with a final protein concentration of 2.5 mg/mL. Data from 100 measurements were collected and analyzed with DYNAMICS v.6.10 software (Wyatt Technology).

#### Small angle X-ray scattering assays (SAXS)

Small angle X-ray scattering (SAXS) measurements were performed with *apo*-BthTX-I and BthTX-I/αT samples on the laboratory based SAXS NANOSTAR (Bruker) equipment. *Apo*-BthTX-I was solubilized in ammonium bicarbonate buffer (50 mM, pH 8.0) at concentration of 5 mg/mL. The protein and the ligand were solubilized in this same buffer and pre-incubated for at least 10 min at 1:05 and 1:1 molar ratio of BthTX-I:αT, with a final protein concentration of 2.5 mg/mL. The radiation was generated by a GeniX 3D (Xenocs) (Cu anode, λ = 1.5411 Å), focused by a FOX 3D (Xenocs) optics and collimated by two sets of scatterless slits providing a beam spot size of 0.5 × 0.5 mm at the sample position. In this configuration, a typical flux of 108 photons/s/mm^2^ was obtained. The 2D data was collected on the V Å NTEC-2000 (Bruker) detector. The samples were placed on reusable quartz capillaries of 1.5 mm in diameter, mounted on stainless steel cases. Therefore, the capillaries can be washed and rinsed, allowing the sample and buffer measurements under the same conditions. The sample to detector distance was 0.68 m, giving a q range of 0.016 < q < 0.35 Å ^−1^, where q is the reciprocal space momentum transfer modulus, defined as q = (4π sinθ)/λ (where 2θ is the scattering angle and λ is the radiation wavelength). The 2D SAXS imagens were recorded for 900 s in a total of 17 frames per sample at room temperature (25 °C), the short exposer time for a long period was performed to assess the stability of the samples and radiation damage. The images were integrated with the Fit2D software^53^. Data processing, blank subtraction, and data normalization were performed with the SUPERSAXS software package^34^. Indirect Fourier Transformation (IFT) procedures were performed using the GNOM program^54^. The comparison of the scattering data with the known atomic resolution structures was made using the CRYSOL program^55^.

Two approaches were applied to assess the oligomerization state of BthTX-I samples under different buffer conditions. In approach (i) the OLIGOMER program^35^ was applied assuming a final scattering profile composed of a mixture of three oligomerization states (monomer, dimer and tetramer) generated by symmetry from the crystallographic structure of PDB id code 3CXI. This approach assumes a system composed of several components and calculates the scattering intensity (${I\left(q\right)}_{total}$) as a linear combination of these components:

1 $$I{\left(q\right)}_{total}=\sum_{i=1}^{N}{\nu }_{i}I{\left(q\right)}_{i}$$

2 $$\sum_{i=1}^{N}{\nu }_{i}=1$$

were ${\nu }_{i}$ is the volume fraction of component *i*, $I{\left(q\right)}_{i}$ is the scattering intensity of component i and N is the total number of components. From the fitting of an experimental data is possible to estimate the fractions of each component that minimize the discrepancy of the model and the experimental SAXS curves. For the approach (ii) the inclusion of aggregates on the modeling assumes an organization of linear monomer arrangements in one, two or N subunits of the monomer from the same crystallographic structure. In this case, the total SAXS intensity resulting from the mixture of particles is described by Eqs. (3) and (4), where the P(q) is the form factor calculated for the monomer using the program CRYSOL^55^ and S(q) is the structure factor which was performed using the Debye equation (Eq. 5) to generate the factor of the aggregated structure where D_ij_ is the distance between the centers of molecules *i* and *j*^56^. The aggregates are considered to be linear with *N* = 2 for the dimers and *N* = 4 for the tetramers. In both cases, volume fractions can be obtained for the populations of aggregates in the system.

3 $${I\left(q\right)}_{total}=P\left(q\right){S}_{total}\left(q\right)$$

4 $${S}_{total}\left(q\right)={f}_{mon}+{f}_{dim}{S}_{dim}(q)+{f}_{tetr}{S}_{tetr}(q)$$

5 $$S\left(q\right)= \sum_{i,j}^{N}\frac{\mathrm{sin}\left(q{D}_{ij}\right)}{{qD}_{ij}}$$

6 $${f}_{mon}+{f}_{dim}+{f}_{tetr}=1$$

### Molecular simulations

#### Setting the systems

The crystallographic atomic coordinates of the *apo-*BthTX-I were taken from the Protein Data Bank (https://www.rcsb.org/), deposited under the PDB id code 3CXI^17^, discarding all the small molecules and the ions bound to the protein. The BthTX-I/αT complex was obtained from the same structure using the same protocol but maintaining the coordinates of the αT molecules. According to Euler angles description^27^, this structure was considered to be an active conformation. In order to determine the inactive conformation, the crystallographic structure under the PDB id code 3HZD was considered^9^.

#### Molecular dynamics (MD) simulations protocol

The *apo-*BthTX-I and BthTX-I/αT systems were built using the CHARMM-GUI webserver^57^ and the CHARMM version 36b1^58^ with the CHARMM36 force field^59^. Each system was placed in a cubic box, solvated with 100 mM NaCl, and subjected to a minimization step using the SD and ABNR algorithms in order to accommodate the solvent molecules by constraining harmonically non-hydrogen atoms of the BthTX-I backbone and αT atoms with a force constant of 1 kcal/mol/ Å ^2^, while BthTX-I side-chain atoms were constrained with a force of 0.1 kcal/mol/ Å ^2^. Then the whole system was equilibrated in an NVT ensemble at 300 K using the Velocity Verlet algorithm during 100 ps with a timestep of 0.001 fs, maintaining the harmonic constraints parameters to both BthTX-I and αT molecules. Bonds with hydrogen atoms were constrained using the SHAKE algorithm^60^. Non-bonded interactions between the atoms were calculated up to 10 Å of distance, with a switching function applied between 10 and 12 Å. All the simulations were carried out under constant temperature and pressure (CPT) ensemble at 300 K and 1 bar using the Nose–Hoover piston^61,62^. The protonation states were calculated by using the PROPKA webserver^63^.

Long MD simulations were also performed to verify the dimeric stability of *apo-*BthTX-I and BthTX-I/αT using GROMACS v.5.0.5 software^64^. This software was used to save computational time, but we maintained the same force field parameters. Each system was placed in a triclinic box, solvated with 100 mM NaCl and minimized to reach an energy gradient below 100 kJ/mol/nm^2^ using the Steepest Descent algorithm. The equilibration steps were performed at 300 K and 1 bar constraining the BthTX-I backbone atoms during 1 ns and then, free MD simulations of 10 or 100 ns were performed for each system using the Nose–Hoover thermostat^61,62^ and Parrinello-Rahman barostat^65^. Non-bonded interactions were calculated applying a switching function for atoms between 10 and 12 Å. The αT topology was converted to GROMACS format using CGenFF^66^.

### Normal modes analysis

Large conformational changes in proteins are directly related to their function. These changes generally occur in a nanosecond to microsecond timescale, which sometimes are not achieved in classical MD. To overcome this limitation, the low-frequency normal mode analysis is an important tool to describe such conformational changes and have been used successfully to describe the structural aspects of many proteins^67^.

Before calculating the normal modes, each protein (with and without the ligand) was energy minimized in vacuum conditions (dielectric constant = 1/r) to a low energy gradient of 10^−6^ kcal × mol × Å ^−1^ using the ABNR algorithm. For each of the energy minimum structures 100 lowest frequency normal modes were calculated using the DIMB method^68^ in CHARMM under the same force field parameters as those described previously. In order to study the large amplitude movements, the eight lowest frequencies modes (numbered from 7 to 14) were selected based on an average Cα atoms fluctuation above 0.1 Å at a temperature of 300 K, and by visual inspection of the structural changes of the protein. These eight modes were used in the MDeNM calculations.

### Molecular dynamics with excited normal modes (MDeNM)

MDeNM is a method consisting of kinetically propagating the conformational changes along a linear combination of a set of normal mode vectors calculated for the system of interest so that these changes can be favored in short MD simulations that take into account the whole system (protein, water molecules, ions, etc.)^36^. A large number of combinations of selected NM vectors are considered ensuring that they are isotropically distributed in order to explore the selected NM space extensively and consequently to generate a well-distributed conformational set. Such exploration would require enormous simulation time if only standard MD simulations were being carried out for molecular systems of the size considered in this work. The appropriate uniform spatially well distributed linear combinations of NM vectors were obtained based on the criterion that the geometrically displaced structures by a given RMSD distance from the initial structure along these vectors are separated from each other by an almost equal RMSD distance. Each MD simulation along a given NM combination vector was called a replica. In this work a replica simulation consisted of a series of 10 kinetic excitations from the equilibrated structure, each excitation corresponded to an increase in the temperature of the whole system of 3 K. As this excess kinetic energy dissipates quickly in a period of approximately 1 ps^36^, the system was excited many times with the interval between two successive excitations being fixed at 5 ps, allowing the system to relax. The structures generated by all the replica simulations were gathered in a first set, called set 1. Each of these structures was subjected to an additional relaxation of 50 ps by free MD simulation to release all the remaining excitation energy. The structures were saved every 1 ps to be analyzed. For each *apo*-BthTX-I and BthTX-I/αT system, 100 replicas were achieved starting from a structure characterized as being in an active state conformation (see above).

The structures of set 1 were analyzed according to the Euler angle analysis^27^ in order to determine the dispersion of the structures around the “active” and inactive states. In this analysis dimeric bothropic PLA_2_-like crystallographic models may be characterized in Euler angles considering the axis of the largest helix as X (roll angle), the vector connecting the two antiparallel helices as Y and the orthogonal axis of the XY plane as Z (tilt)^27^. Inactive PLA_2_-like dimer possesses void hydrophobic channel and asymmetrical monomers with roll and tilt as 141° (± 0.6°) and 54° (± 1.0°), respectively^27^. The active has the hydrophobic channel occupied by a hydrophobic molecule, has symmetrical monomers with both Leu121 and Phe125 in a 3_10_ helix conformation with roll and tilt as 174° (± 5.5°) and 37° (± 15.4°), respectively^27^.

The combined NM vector that best described the movement from the active to the inactive state was used for additional MDeNM excitation steps starting from the same starting structure as before but using different initial conditions to approach closer to the inactive state, resulting into structure set 2. For this set, 10 replicas were generated for the *apo-*BthTX-I and BthTX-I/αT systems using the selected vector, each one composed of 20 excitations to ensure that the protein reached the inactive state. Subsequently, the structures of each system that had the lowest RMSD from the inactive structure (PDB id 3HZD^9^) were submitted to a free MD simulation of 10 ns using GROMACS 5.0.5 software under the same energy parameters previously described in order to analyze the stability of the found conformation, resulting in structure set 3.

### Structural analysis

Different structural characteristics of BthTX-I were analyzed, including: (i) distance between the center of masses of Helix-I and MDiS, the former covering the residues 1–4, and the second the residues 111–114 as indicated in Supplementary Fig. S6; (ii) distribution of the roll and tilt Euler angles (Supplementary Fig. S7A) and the distance of MDiS from the sulfate plane (Supplementary Fig. S7B), as described previously by Borges and cols.^27^; (iii) percentage of atomic contacts of BthTX-I with αT, with a contact being defined when the distance between the respective non-hydrogen atoms is less than 4.5 Å; (iv) other quantities, such as the backbone RMSD from the starting structure, radius of gyration (R_g_), solvent-accessible surface area (SASA), and normal mode fluctuations. Both GOMACS or CHARMM software were used to perform these calculations.

## Supplementary information

Supplementary Information 1

Supplementary Information 2
