# Supplementary material for: The allosteric activation mechanism of a phospholipase A2-like toxin from Bothrops jararacussu venom: a dynamic description
Source: Sci Rep. 2020 Oct 1;10:16252. doi: 10.1038/s41598-020-73134-9 (PMC7529814; doi:10.1038/s41598-020-73134-9)
Supplement: Supplementary file 2 — Supplementary Information 2 [file 41598_2020_73134_MOESM2_ESM.docx]

The allosteric activation mechanism of a phospholipase A_2_-like toxin from *Bothrops jararacussu* venom – a dynamic description

Antoniel A. S. Gomes^1,2^, Fabio F. Cardoso^1^, Maximilia F. Souza^3^, Cristiano L. P. Oliveira^3^, David Perahia^2^, Angelo J. Magro^4,5^*, Marcos R. M. Fontes^1,^*

**SUPPLEMENTARY MATERIAL**


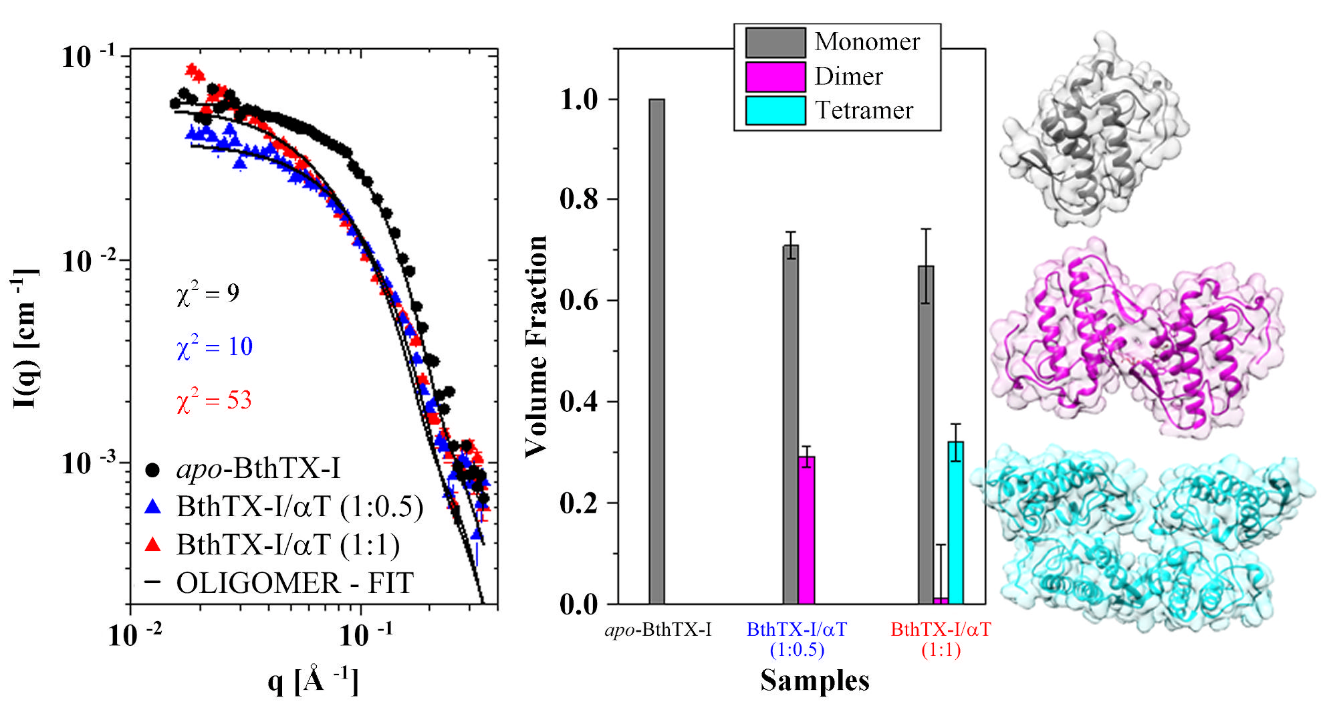


**Supplementary figure S1.** Applying a mixture of BthTX-I oligomerization to describe the SAXS experimental data to *apo*-BthTX-I (black symbols) and BthTX-I/αT in solution with a molar ratio of 1:0.5 (blue symbols) and 1:1 (red symbols). The optimization fit is shown in black line; the volume fraction to each model that best describe the respective SAXS data is shown on the right as well as the structures models applied as an input to OLIGOMER program^35^.

**
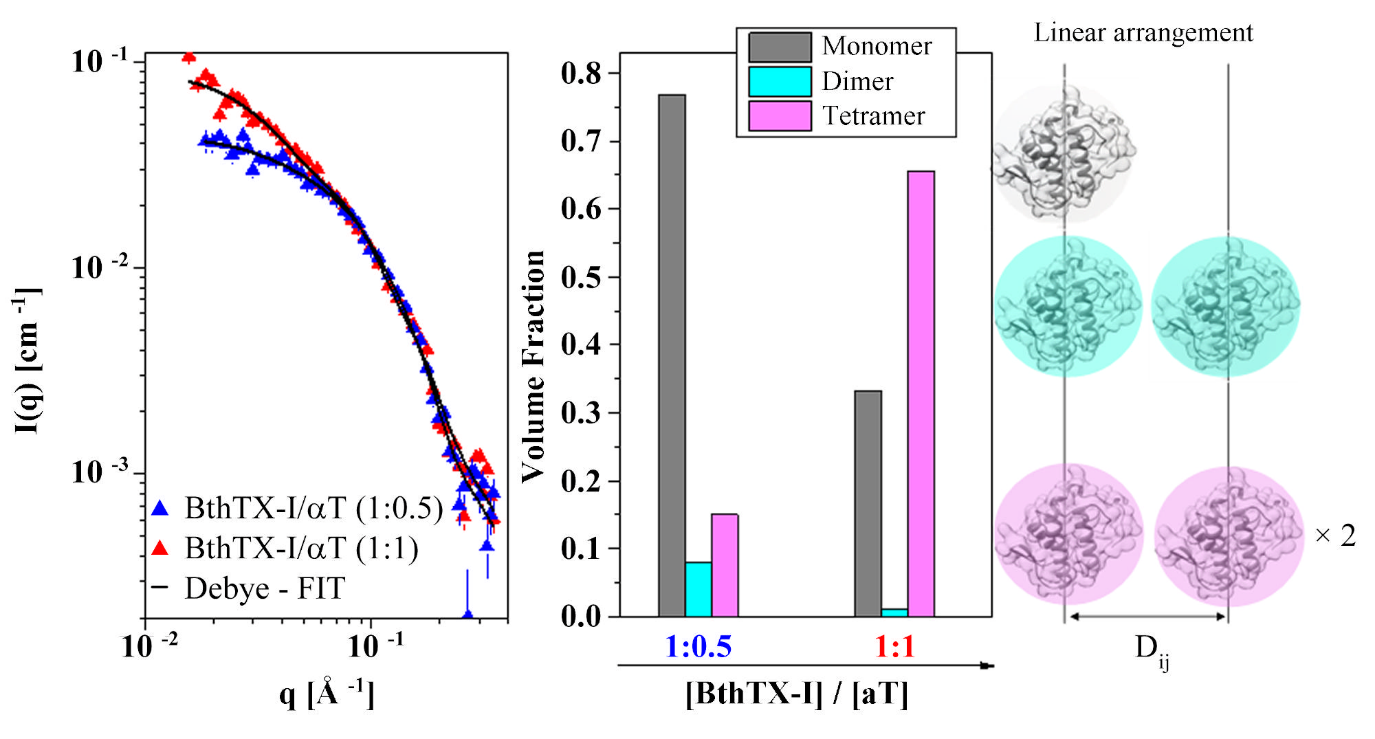
**

**Supplementary figure S2.** Applying a mixture of BthTX-I oligomerization to describe the SAXS experimental data to BthTX-I/αT in solution with a molar ratio of 1:0.5 (blue symbols) and 1:1 (red symbols). The optimization fit is shown in black line; the volume fraction to each model that best describe the respective SAXS data is shown on the right as well as one example of the linear arrangement of the monomeric structure to form dimers and tetramers applying Debye approach.

**
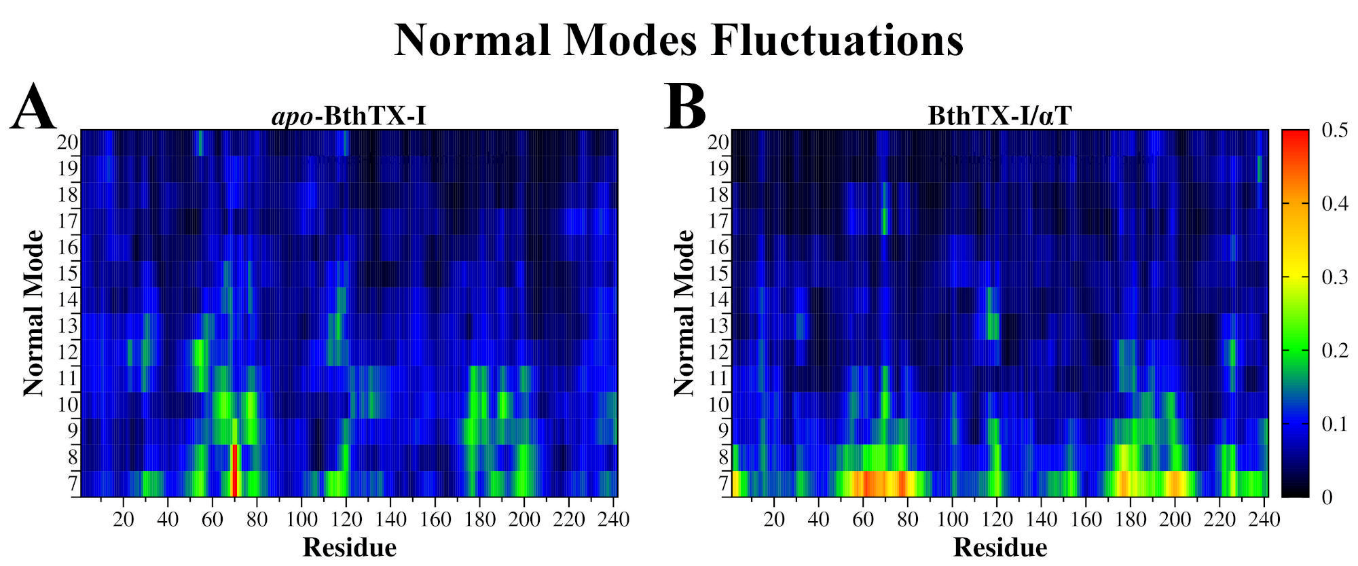
**

**Supplementary figure S3.** Normal modes fluctuation of the Cα atoms as a function of residues for the *apo*-BthTX-I (**A**) and BthTX-I/αT (**B**) systems. The fluctuation levels, in nanometers, are presented in colors ranging from black-blue (bottom) to orange-red (top), as shown in the palette on the right. The residues of the BthTX-I dimer were arranged in sequence, resulting in 242 residues (121 residues for each monomer).


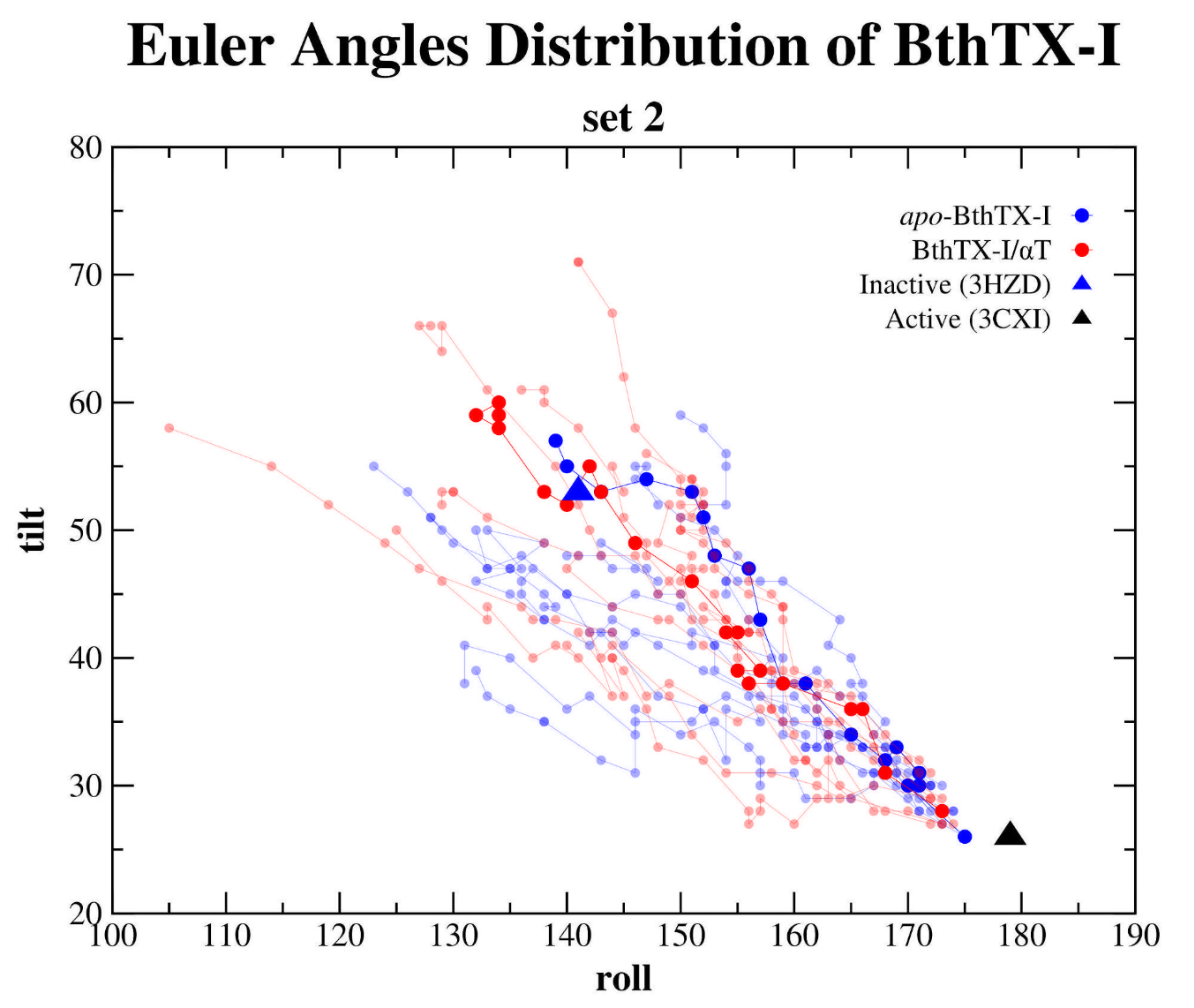


**Supplementary figure S4.** Distribution of the roll/tilt Euler angles of the *apo*-BthTX-I (blue circles) and BthTX-I/αT (red circles) structures from set 2, represented as transparent filled circles. The completely filled circles for each system relate to the structures resulting from the replica simulation leading to the proximity of the inactive state. The structures related to the same replica are connected by lines. The inactive (PDB id 3HZD) and active (PDB id 3CXI) states are represented by solid triangles in blue and black, respectively.


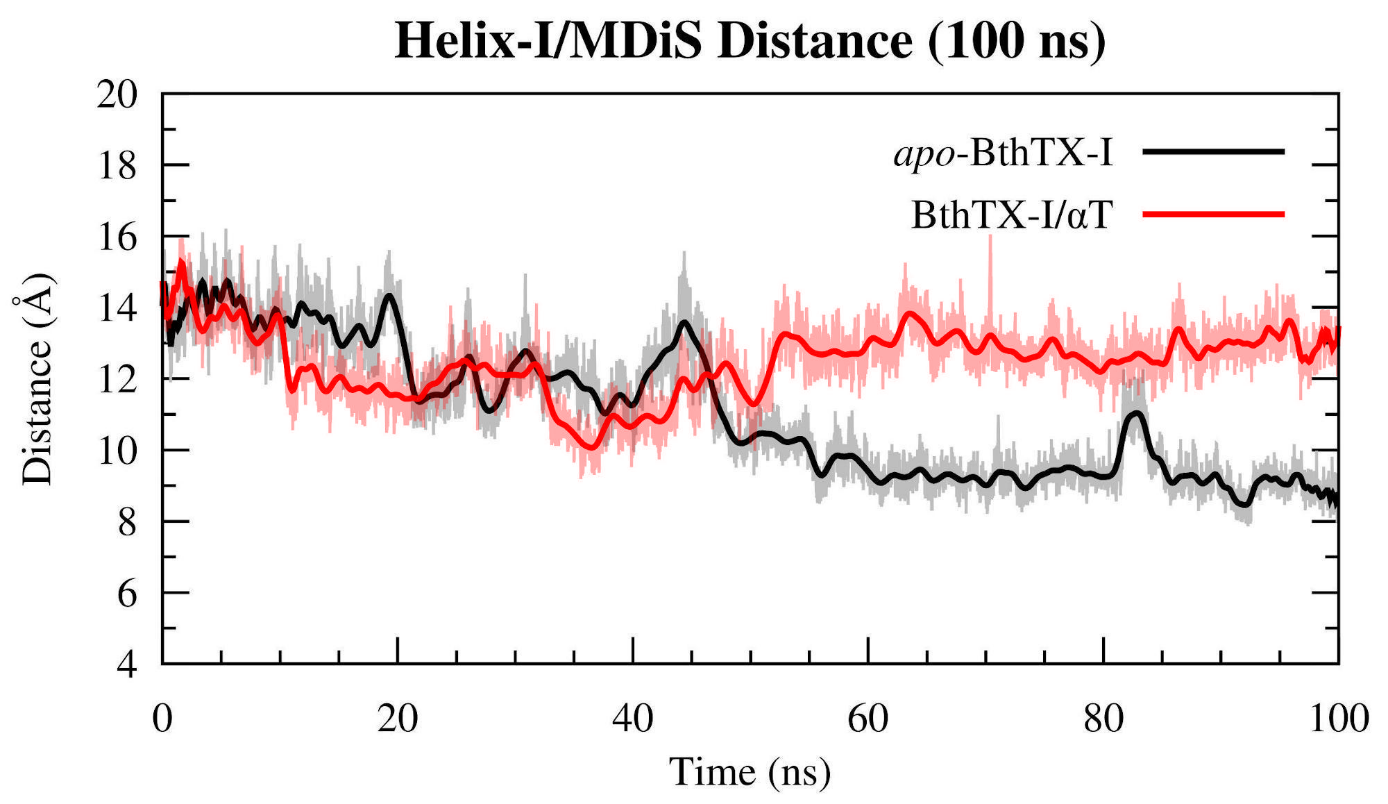


**Supplementary figure S5.** Time evolution of the Helix-I/MDiS distance from the active state during 100 ns of free MD for *apo*-BthTX-I (initial structure from PDB id 3CXI) (black line) and for BthTX-I/αT complex (red line).


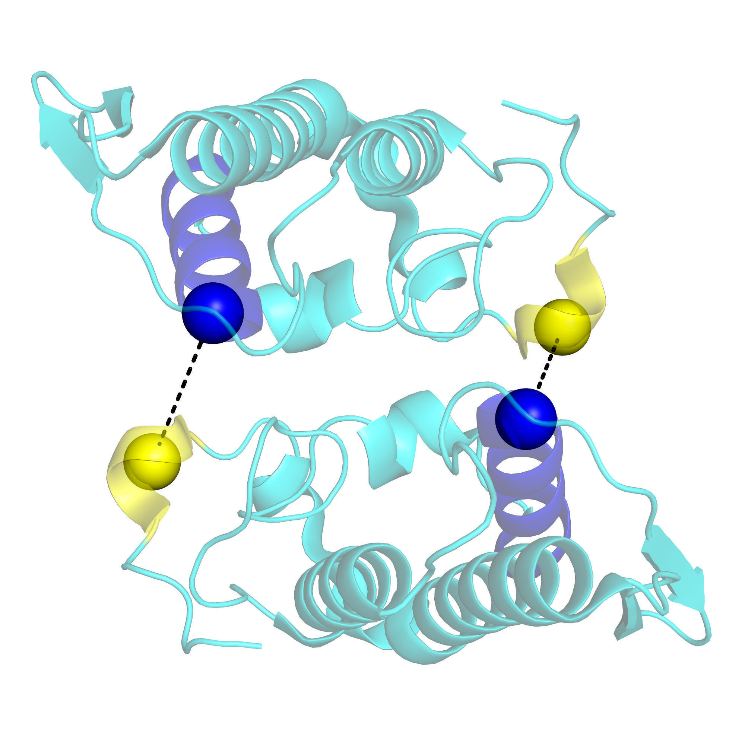


**Supplementary figure S6.** Crystallographic structure (PDB ID 3CXI) of the BthTX-I dimer (cyan), highlighting Helix-I (blue) and the MDiS domain (yellow). The Helix-I/MDiS distance is calculated by the distance (dotted lines) of the center of mass of the region defined by the residues from Ala1 to Tyr4 for Helix-I (blue spheres), and that from Leu111 to Phe114 residues (yellow spheres) for each subunit.


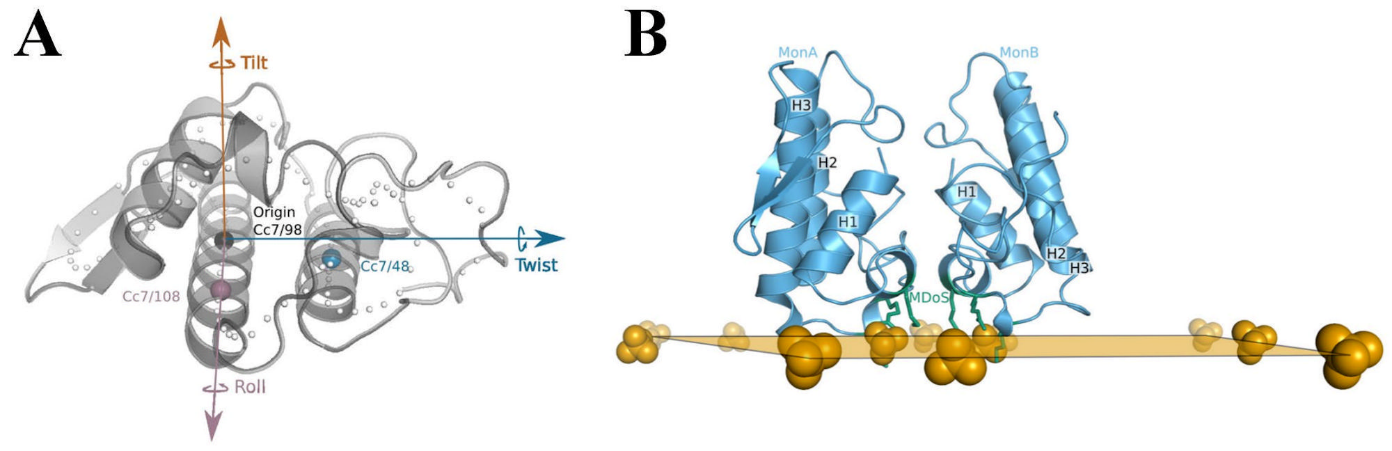


**Supplementary figure S7.** **A.** Roll, tilt and twist Euler angles of BthTX-I strucure, and **B.** The sulfate plane generated from crystallographic sulfates, as described by Bahnson^37^. The co-crystallized sulfate ions of a dimeric PLA_2_ protein are oriented as a plane which mimics the phospholipid heads of biological membranes; therefore, their coordinates were used to build the sulfate plane. Both panels were obtained from Borges *et al*.^27^.

**Supplementary table S1.** Structural parameters from SAXS data: radius of gyration (R_g_), maximum diameter (D_max_) and molecular weight (MW).


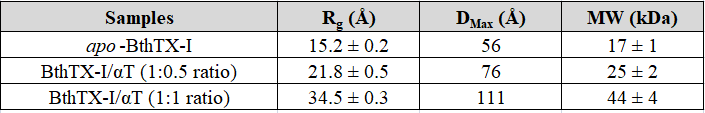


**Supplementary table S2**. Percentage of contacts between non-hydrogen atoms of each residue of *apo-*BthTX-I and BthTX-I/αT structures from sets 1 and 3. A contact is defined when at least one of the interatomic distances is below 4.5 Å.


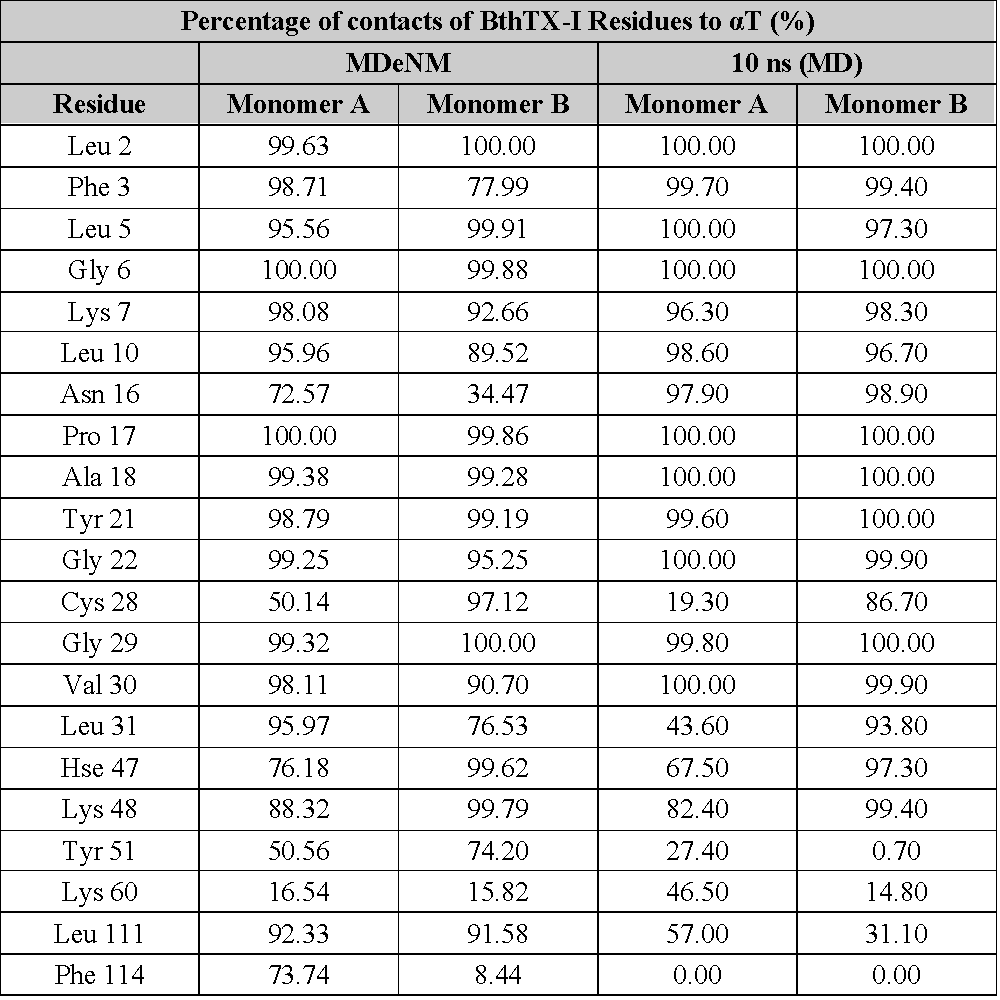


**Supplementary video S1.** The activation process of BthTX-I (cyan), showing the approximation of the MDiS region (yellow) to the membrane. The MDiS Leu111 and Phe114 residues are shown as yellow sticks, and the phospholipid heads (phosphates) of the membrane are shown as sticks.
